# EHMTI-0372. No abnormalities of intrinsic brain connectivity during the interictal phase of migraine with aura

**DOI:** 10.1186/1129-2377-15-S1-K5

**Published:** 2014-09-18

**Authors:** A Hougaard, FM Amin, S Magon, T Sprenger, E Rostrup, M Ashina

**Affiliations:** 1 Danish Headache Center and Faculty of Health and Medical Sciences, Glostrup Hospital and University of Copenhagen, Copenhagen, Denmark; 2 Department of Neurology, University Hospital Basel, Basel, Switzerland; 3 Functional Imaging Unit and Faculty of Health and Medical Sciences, Glostrup Hospital and University of Copenhagen, Copenhagen, Denmark

## Introduction

Functional neuroimaging studies have shown hyperresponsiveness of cortical areas to visual stimuli in migraine patients with aura outside of attacks. This may be a key feature in the initiation of aura episodes and possibly also migraine headache attacks. It is unknown if cortical dysfunction is present at rest, i.e. in the absence of any external stimuli. Functional magnetic resonance imaging (fMRI) is a powerful technique for evaluating resting-state functional connectivity, i.e. coherence of brain activity across cerebral areas.

## Aims

To investigate resting-state functional brain connectivity in migraineurs with aura outside of attacks using fMRI.

## Methods

We investigated 40 patients suffering from migraine with visual aura and 40 individually age- and gender-matched healthy controls with no history or family history of migraine. Following advanced denoising, the data were analyzed both in a hypothesis-driven fashion, testing for abnormalities involving 27 different brain areas of potential relevance to migraine with aura, including the cortical visual areas, the amygdala and peri-aqueductal grey matter, and in a data-driven, exploratory fashion (dual regression) in order to reveal any possible between-group differences of resting state networks. Age, gender, attack frequency, and disease duration were included as nuisance variables.

## Results

We found no differences of functional connectivity between patients and controls.

## Conclusions

The previously reported increased cortical hyperresponsivity in the interictal phase of migraine with aura is unlikely to be caused by abnormalities of intrinsic brain connectivity. The interictal migraine aura brain may be abnormally functioning only during exposure to external stimuli.

No conflict of interest.

